# Utilizing a TLR5-Adjuvanted Cytomegalovirus as a Lentiviral Vaccine in the Nonhuman Primate Model for AIDS

**DOI:** 10.1371/journal.pone.0155629

**Published:** 2016-05-16

**Authors:** Jesse D. Deere, W. L. William Chang, Luis D. Castillo, Kim A. Schmidt, Hung T. Kieu, Nicholas Renzette, Timothy Kowalik, Stephen W. Barthold, Barbara L. Shacklett, Peter A. Barry, Ellen E. Sparger

**Affiliations:** 1 Center for Comparative Medicine, University of California Davis, Davis, California, United States of America; 2 Department of Veterinary Medicine and Epidemiology, School of Veterinary Medicine, University of California Davis, Davis, California, United States of America; 3 Department of Microbiology and Physiological Systems, University of Massachusetts Medical School, Worcester, Massachusetts, United States of America; 4 Department of Veterinary Pathology, Microbiology and Immunology, School of Veterinary Medicine, University of California Davis, Davis, California, United States of America; 5 Department of Medical Microbiology and Immunology, School of Medicine, University of California Davis, Davis, California, United States of America; Harvard Medical School, UNITED STATES

## Abstract

Despite tremendous progress in our understanding of human immunodeficiency virus (HIV) natural history and advances in HIV treatment, there is neither an approved vaccine nor a cure for infection. Here, we describe the development and characterization of a novel replicating vaccine vector utilizing Cytomegalovirus (CMV) and a TLR5 adjuvant. After partial truncation of the central, immunodominant hypervariable domain, flagellin (*fliC*) from Salmonella was cloned downstream of a codon optimized *gag* gene from simian immunodeficiency virus (SIV) and transiently expressed in telomerized rhesus fibroblast (TeloRF) cells in culture. Lysates generated from these transfected cells induced the tumor necrosis factor alpha (TNF-α), in a mouse macrophage cell line, in a TLR5-dependent manner. The Gag/FliC expression construct was cloned into a bacterial artificial chromosome encoding the rhesus CMV (RhCMV) genome, and infectious RhCMV was generated following transfection of TeloRF cells. This virus stably expressed an SIV Gag/FliC fusion protein through four serial passages. Lysates generated from infected cells induced TNF-α in a TLR5-dependent manner. Western blot analysis of infected cell lysates verified expression of a Gag/FliC fusion protein using a SIV p27 capsid monoclonal antibody. Lastly, rhesus macaques inoculated with this novel RhCMV virus demonstrated increased inflammatory responses at the site of inoculation seven days post-infection when compared to the parental RhCMV. These results demonstrate that an artificially constructed replicating RhCMV expressing an SIV Gag/FliC fusion protein is capable of activating TLR5 in a macrophage cell line *in vitro* and induction of an altered inflammatory response *in vivo*. Ongoing animals studies are aimed at determining vaccine efficacy, including subsequent challenge with pathogenic SIV.

## Introduction

Thirty years of research has revealed major insights into the human immunodeficiency virus (HIV-1) replication cycle, pathogenesis, and immune responses; however, there is neither an approved vaccine nor a broadly applicable cure for HIV-1 infection. Although combination antiretroviral therapy (cART) [1] is capable of controlling HIV-1 infection, low-level viremia persists [2] and virus in plasma rebounds if cART is interrupted [3]. HIV-1 infected people must also remain on cART indefinitely to prevent further destruction of their immune systems and progression to acquired immune deficiency syndrome (AIDS). Chronic, non-AIDS associated diseases in people undergoing long-term treatment demonstrate that cART does not completely restore health and emphasize the need for a vaccine or a cure (reviewed in [4, 5]).

Results from the RV144 vaccine clinical trial suggest for the first time that a vaccine to prevent the acquisition of HIV-1 might be possible. The RV144 vaccine regimen consisted of four serial vaccinations with a canarypox vector expressing the HIV-1 Envelope glycoprotein (gp120; ALVAC-HIV) combined with two serial vaccinations with recombinant HIV-1 gp120 (AIDSVAX B/E) [6]. Modified intention-to-treat analysis suggested a 31% vaccine efficacy in prevention of HIV-1 acquisition in the vaccine group versus the placebo control group for the three-year study duration [6]. However, a follow-up study revealed a cumulative vaccine efficacy in prevention of HIV-1 infection of 61% through the first 12 months after immunization that declined over time [7]. While the decline in vaccine efficacy over time requires further investigation to fully understand the mechanism, additional or prolonged boosting of the initial immune response might be required to improve long-term vaccine efficacy. It is important to note that while the RV144 vaccine regimen reduced the risk of HIV-1 infection, it did not reduce viral load or protect from loss of CD4+ T cells in those that became infected [6].

A unique vaccination strategy utilizing recombinant viral vectors takes advantage of the persistent nature of the betaherpesvirus, cytomegalovirus (CMV). Using the well-defined rhesus macaque (*Macaca mulatta*) nonhuman primate model of AIDS, recombinant rhesus CMVs (RhCMV) have been constructed that express the simian immunodeficiency virus (SIV) proteins: Gag, Env, and Rev/Tat/Nef (and Pol) [8, 9]. These RhCMV-vectored SIV vaccines induced effector T cell immune responses that resulted in long-term control of SIV infection in 50% of the mucosally-challenged animals [9], with some animals apparently clearing the infection [10]. Prolonged antigenic stimulation due to the persistent nature of the RhCMV vector combined with an unconventional CD8 T-cell response suggest novel, unprecedented protective mechanisms that could confer long-term control and clearance of virulent SIV infection [8, 11]. Whether these responses can be enhanced to protect a greater percentage of the challenged animals is key to further development of this promising strategy.

One method of enhancing immunogenicity of a particular antigen is through the use of an adjuvant. Pattern recognition receptor agonists, including the Toll-like receptor 5 (TLR5) agonist, Flagellin (FliC), have been tested as vaccine adjuvants that manipulate the innate immune response and thereby strengthen adaptive immune responses to particular antigens (reviewed in [12, 13]). Recent studies have demonstrated enhanced immunogenicity of recombinant antigens delivered either concomitantly or fused with FliC in mice and nonhuman primate animal models [14–20]. Stimulation of the TLR5 receptor by FliC resulted in the induction of a proinflammatory response and the direct activation of TLR5-expressing CD11c+ dendritic cells [12, 21]. FliC may also directly activate CD4+ and CD8+ lymphocytes (reviewed in [12]).

Here, we describe the construction of a recombinant RhCMV that expresses a SIV Gag-Salmonella FliC fusion protein in which the region of FliC containing immunodominant epitopes has been deleted. This virus is subsequently characterized for expression of an SIV Gag FliC fusion protein with TLR5 ligand-specific bioactivity.

## Results

### FliC hypervariable region mutants stimulate TNF-α Expression *in vitro*

The two TLR5 binding domains of FliC are located within the well-conserved N and C termini (amino acid residues 79–117 and 408–439, respectively) [22]. The central region consists of an immunodominant hypervariable (HV) domain (residues 185–306) that is targeted by the adaptive immune system [22]. The HV domain of FliC was partially truncated to prevent an adaptive immune response to the adjuvant portion of the vaccine and to reduce the size of the total fusion open reading frame for more efficient cloning into the RhCMV bacterial artificial chromosome (BAC) [23]. Two separate *Salmonella enterica* serovar Enteritidis *fliC* truncations were constructed by deleting the nucleotides encoding amino acid residues 196–378 (*fliC*Δ196–378), as previously described [14], in addition to a novel second, larger deletion of amino acid residues 141–398 (*fliC*Δ141–398). These genes, along with wild-type *fliC*, were cloned into the eukaryotic expression vector, pORI, under control of the EF1 alpha promoter, generating the three plasmids: pORI*fliC*Δ196–378, pORI*fliC*Δ141–398 and pORI*fliC*.

**Fig 1:**
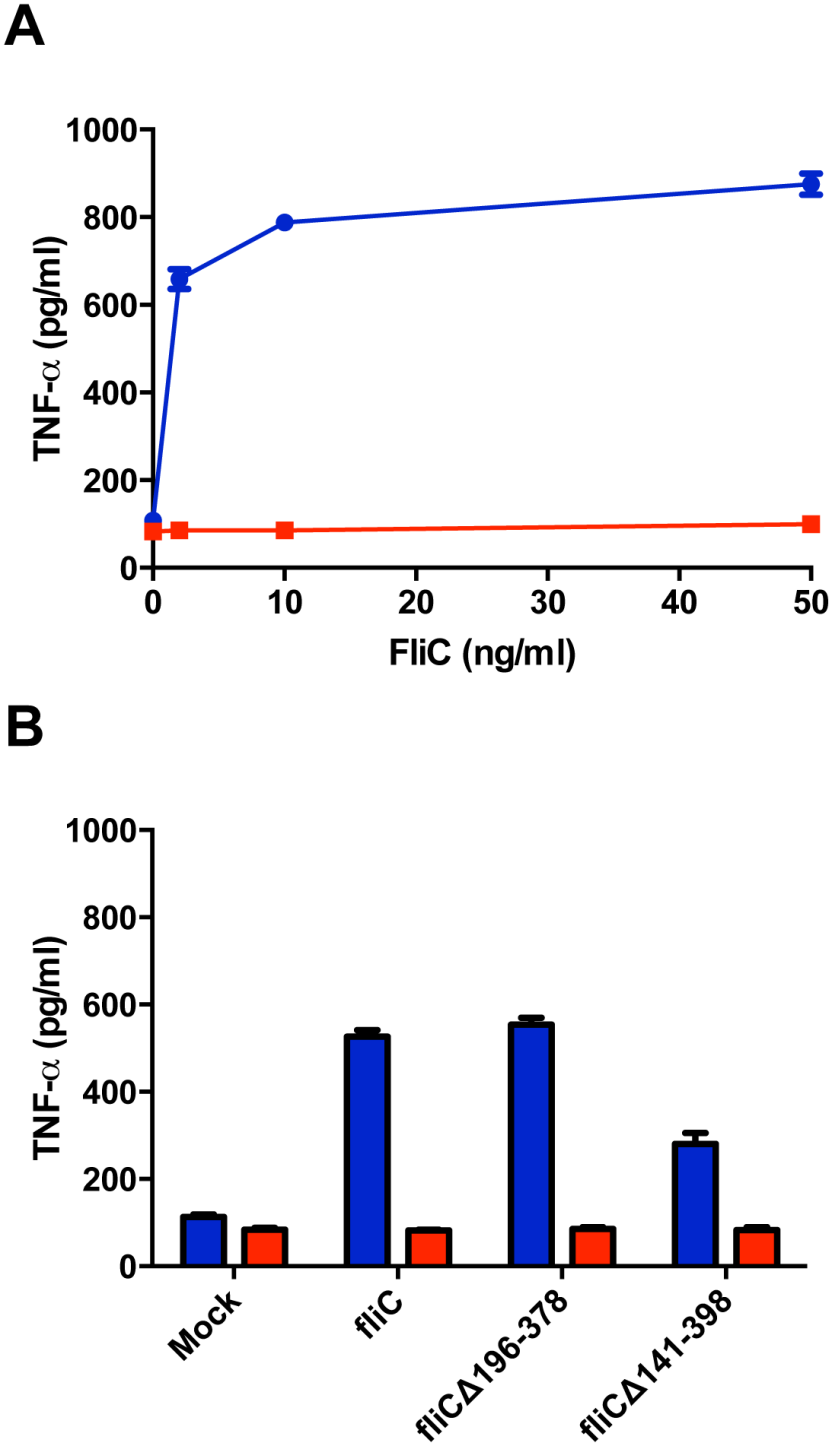
Recombinant FliC and truncated FliC expressed from plasmid pORI induce TNF-α in a TLR5-dependent manner. TNF-α ELISA of cell culture supernatants collected from TLR5-positive RAW424 (Blue) or its parental TLR5-negative RAW264.7 (Red) mouse macrophage cells. **(A)** RAW cells were treated with increasing concentrations of recombinant FliC. **(B)** Telomerized rhesus fibroblast cells were transiently transfected with plasmids containing full-length FliC (pORI*fliC*) or partially truncated FliCs (pORI*fliC*Δ196–378 or pORI*fliC*Δ141–398) and freeze thaw lysates were generated. Cell culture supernatants harvested from RAW cells treated with these lysates were assayed by an ELISA that measures TNF-α production. An average of two independent experiments and means with standard deviation are presented.

The TLR5-positive mouse macrophage cell line (RAW424) and its parental TLR5-negative cell line (RAW264.7) [14] were treated with recombinant FliC to measure TLR5-specific bioactivity. Cell culture supernatants were collected from the RAW cells and analyzed by enzyme-linked immunosorbant assay (ELISA) for proinflammatory cytokine, tumor necrosis factor-α (TNF-α). Treatment of RAW424 cells with recombinant FliC confirmed cell line secretion of TNF-α by ELISA, while the absence of TNF-α in FliC-treated RAW264.7 cell supernatants confirmed that the RAW424 cell response was TLR5 specific (Fig 1A).

To determine the TLR5-specific bioactivity of the FliC truncations, plasmids pORI*fliC*Δ196–378, pORI*fliC*Δ141–398 and pORI*fliC* were transfected into telomerized rhesus fibroblast (TeloRF) cells and freeze-thaw lysates were generated. RAW424 cells exposed to these lysates demonstrated that all three plasmids induced TNF-α in a TLR5 dependent manner (Fig 1B). Plasmid pORI*fliC*Δ196–378 stimulated TNF-α levels similar to those observed with wild-type flagellin (pORI*fliC*) (Fig 1B). However, pORI*fliC*Δ141–398 stimulated significantly lower concentrations of TNF-α compared to those induced by plasmids pORI*fliC* and pORI*fliC*Δ196–378 based on a one-way ANOVA with Tukey’s multiple comparisons test (Fig 1B; *P* value < 0.0001). Plasmid pORI*fliC*Δ196–378 was selected for construction of a SIV Gag FliC fusion protein as a vaccine antigen based on these results.

### SIV Gag-FliC fusion proteins stimulate TNF-α *in vitro*

**Fig 2:**
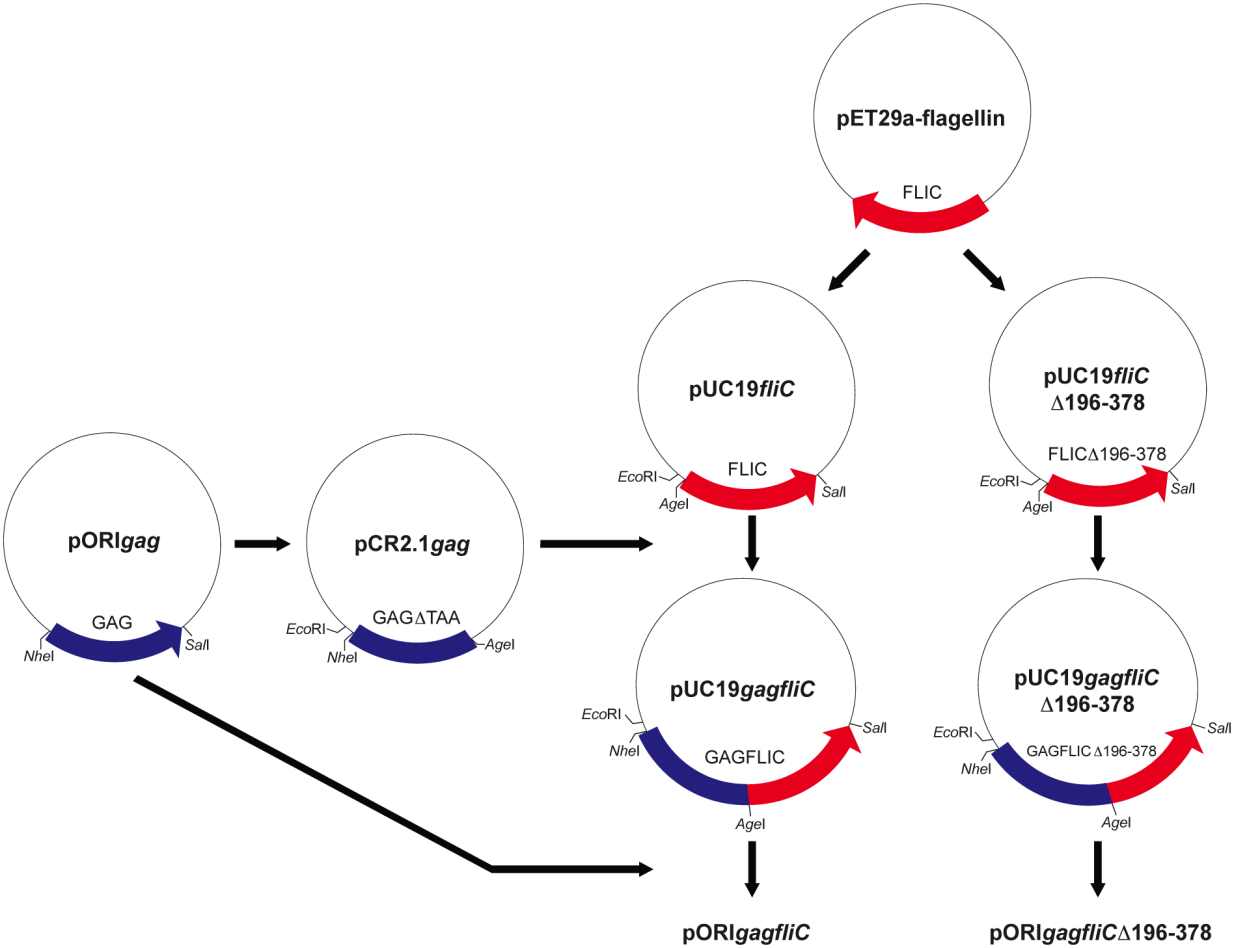
Flow Chart depicting construction of vectors encoding SIV *gag* Salmonella *fliC* fusion proteins. Salmonella *fliC* was cloned in frame downstream of the SIV *gag* gene. The final fusion construct encoded a single Kozak sequence and translation start codon at the 5’ end, and a single stop codon at the 3’ end. A single AgeI restriction endonuclease recognition site separated the two proteins.

**Fig 3:**
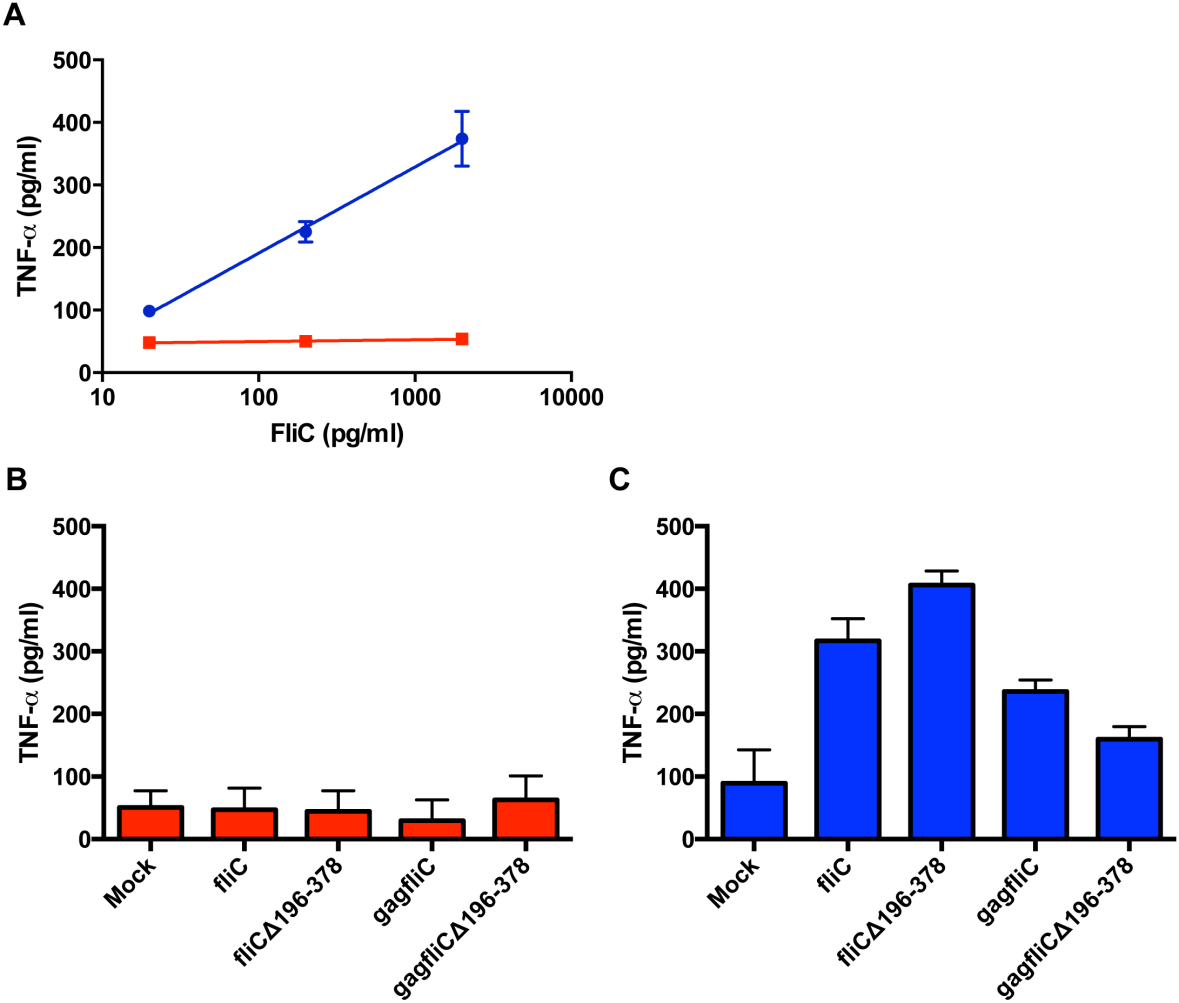
Gag-FliC and GagFliCΔ196–378 fusion proteins induce TNF-α in a TLR5-dependent manner. TNF-α ELISA analysis of cell culture supernatants collected from TLR5-positive RAW424 cells (Blue) or TLR5-negative RAW264.7 cells (Red) are shown. **(A)** Linear regression analysis of RAW cells treated with recombinant FliC. **(B)** RAW264.7 cells or **(C)** RAW424 cells treated with freeze/thaw lysates collected from TeloRF cells transiently transfected with the expression vectors pORI*fliC*, pORI*fliC*Δ196–378, pORI*gagfliC* or pORI*gagfliC*Δ196–378. Data represent the average of two independent experiments with means and standard deviations shown by error bars.

Previous studies have demonstrated that antigens directly fused to FliC induce superior immune responses compared to delivery of vaccine antigen and FliC as separate proteins [14, 21]. Plasmids encoding SIV Gag-FliC fusion proteins were constructed and assessed for induction of TNF-α. SIV *gag* was cloned into the expression vector, pORI, and two separate expression plasmids were constructed by inserting either wild type *fliC* or *fliC*Δ196–378 downstream of the SIV *gag* gene (full length), generating pORI*gagfliC* and pORI*gagfliC*Δ196–378 (Fig 2). A single AgeI restriction endonuclease recognition site (REase) separated the SIV *gag* and *fliC* genes. The resulting fusion genes encoded a single Kozak sequence and a single start codon located at the 5’ end of SIV *gag*, and a single transcription termination signal located at the 3’ end of *fliC* or *fliC*Δ196–378. Cellular lysates were generated from TeloRF cells transiently transfected with SIV gag/fliC expression plasmids to assess for TLR5 stimulating activity. RAW424 (TLR5^+^) and RAW264.7 (TLR5^-^) cells were treated with the lysates, and cell culture supernatants were analyzed for concentrations of TNF-α by ELISA. Treatment of RAW cells with a 3-log_10_ serial dilution of recombinant FliC demonstrated the linear range of the assay while the lack of a response in RAW264.7 cells treated with recombinant FliC demonstrated that the observed TNF-α stimulation in treated RAW424 cells was dependent upon TLR5 (Fig 3A). RAW264.7 cells treated with lysates from TeloRF cells transfected with any of the FliC encoding plasmids did not result in induction of TNF-α (Fig 3B). In contrast, RAW424 cells treated with the same TeloRF lysates exhibited significant induction of TNF-α over mock-treated RAW424 cells (Fig 3C). A one-way ANOVA with Tukey’s multiple comparisons test determined that the observed levels of TNF-α induced by each FliC-encoding vector was significantly different from the RAW424 cells treated with lysates from mock-transfected TeloRF cells (*P* value < 0.0001). Together, these data demonstrated that SIV Gag-FliC and SIV Gag-FliCΔ196–378 fusion proteins expressed from transfected TeloRF cells induce TNF-α in RAW424 cells in a TLR5-dependent manner.

### RhCMV encoding an SIV Gag-FliCΔ196–378 fusion protein stimulates TNF-α *in vitro*

**Fig 4:**
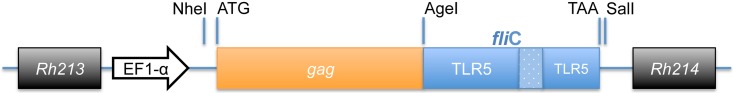
RhCMV*gagfliC*Δ196–378 virus map. The SIV *gag*-Salmonella *fliC* fusion gene was cloned from the expression plasmid pORI*gagfliC*Δ196–378 into a BAC encoding the RhCMV 68–1 genome. The insertion site was in the intergenic region between Rh213 and Rh214 of the RhCMV genome. The open arrow indicates the EF1-α promoter. The start and stop codons and the restriction endonuclease sites used in the cloning process are indicated above the colored bars. The orange bar indicates SIV *gag*. The solid blue bars designate the conserved carboxyl and amino termini of *fliC*, containing the TLR5 binding domains. The light blue bar with white dots designates the partially truncated *fliC* hypervariable domain.

**Fig 5:**
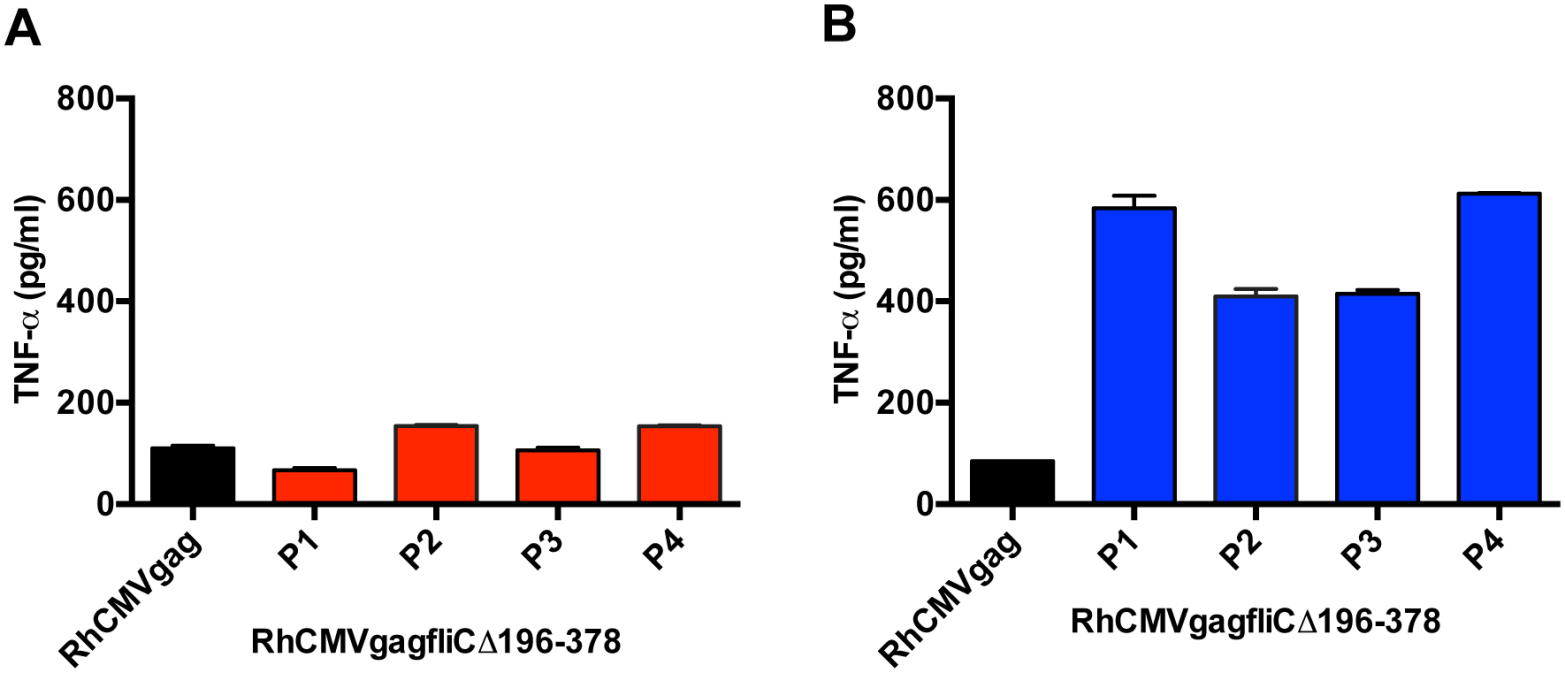
RhCMV*gagfliC*Δ196–378 serial passage in TeloRF cells. RAW264.7 cells **(A)** or RAW424 cells **(B)** were treated with lysates from TeloRF cells infected with RhCMV*gag* (black bars) or RhCMV*gagfliC*Δ196–378 upon serial passage. Cell culture supernatants were collected after four hours of treatment and analyzed for TNF-α by ELISA. Data represent means with standard deviations.

To generate RhCMV expressing an SIV Gag-FliC fusion protein, the SIV *gagfliC*Δ196–378 fusion gene was cloned from pORI*gagfliC*Δ196–378 into a previously described BAC [23] encoding an infectious RhCMV genome (RhCMV 68–1) using Red/ET and Flp-mediated recombination, generating RhCMV*gagfliC*Δ196–378 BAC. The *gagfliC*Δ196–378 insertion site was located in the intergenic region between rh213 and rh214 genes of RhCMV-*loxP*(r) (Fig 4) [8, 23]. TeloRF cells transfected with the RhCMV*gagfliC*Δ196–378 BAC were maintained until the culture exhibited nearly complete cytopathic effect. Cell culture supernatant was collected, designated passage one (P1) virus and used to infect fresh TeloRF cells through four serial passages. Cell culture lysates were generated from each viral passage and analyzed using RAW264.7 cells (Fig 5A) and RAW424 cells (Fig 5B) for induction of TNF-α by ELISA. The results demonstrated that RhCMV*gagfliC*Δ196–378 induced production of TNF-α in a TLR5-dependent manner and that this activation of TLR5 was stable through all four serial passages.

**Fig 6:**
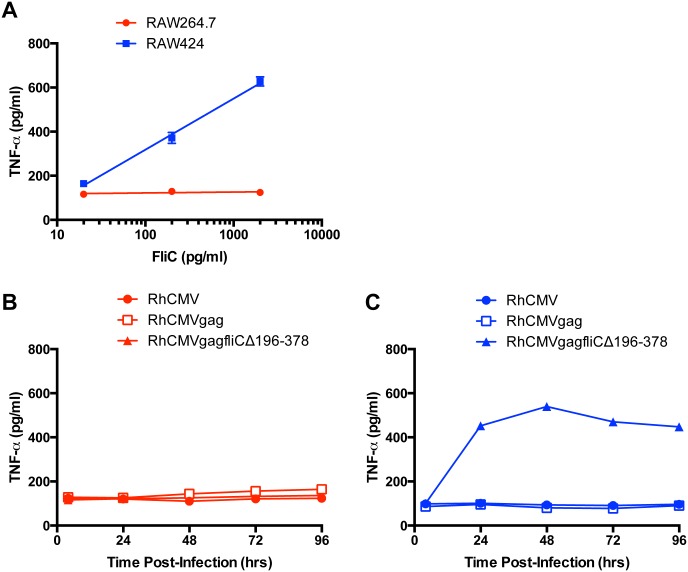
RhCMV-encoded Gag-FliCΔ196–378 fusion protein expression kinetics. **A)** To determine the linear range of the TLR5 Biological Assay, RAW cells were treated with recombinant FliC, and TNF-α in the supernatant was quantified by ELISA. The graph indicates linear regression analysis. **B and C)** TeloRF cells were then infected with RhCMV, RhCMV*gag*, or RhCMV*gagfliC*Δ196–378 and cell lysates were generated at the indicated times post-infection. The infected cell lysates were assessed for induction of TNF-α as described above on RAW264.7 cells **(B)** or RAW424 cells **(C)**.

To determine the kinetics of RhCMV*gagfliC*Δ196–378 gene expression, cell lysates prepared from infected TeloRF cells were assayed for induction of TNF-α on RAW cells as described above. Treatment of RAW cells with recombinant FliC determined the linear range of the assay (Fig 6A). Accordingly, treatment of RAW264.7 cells with the RhCMV*gagfliC*Δ196-378-infected TeloRF cell lysates did not induce TNF-α (Fig 6B) while treatment of RAW424 cells with the same lysates demonstrated increasing TNF-α induction over time, with peak expression levels occurring with the lysates collected at 48 hours post-infection (Fig 6C). The results demonstrated maximum GagFliCΔ196–378 expression from recombinant RhCMV*gagfliC*Δ196–378 within 48 hours of infection in TeloRF cells with gene expression stable for at least 96 hours post-infection (Fig 6C). The absence of TNF-α induction in RAW cells treated with lysates from TeloRF cells infected with either RhCMV or RhCMV expressing SIV Gag alone (RhCMV*gag*) confirmed that induction of TNF-α was specific to the *gagfliC*Δ196–378 insert.

### RhCMV-expressed SIV Gag-FliCΔ196–378 fusion protein remains intact in TeloRF cells

**Fig 7:**
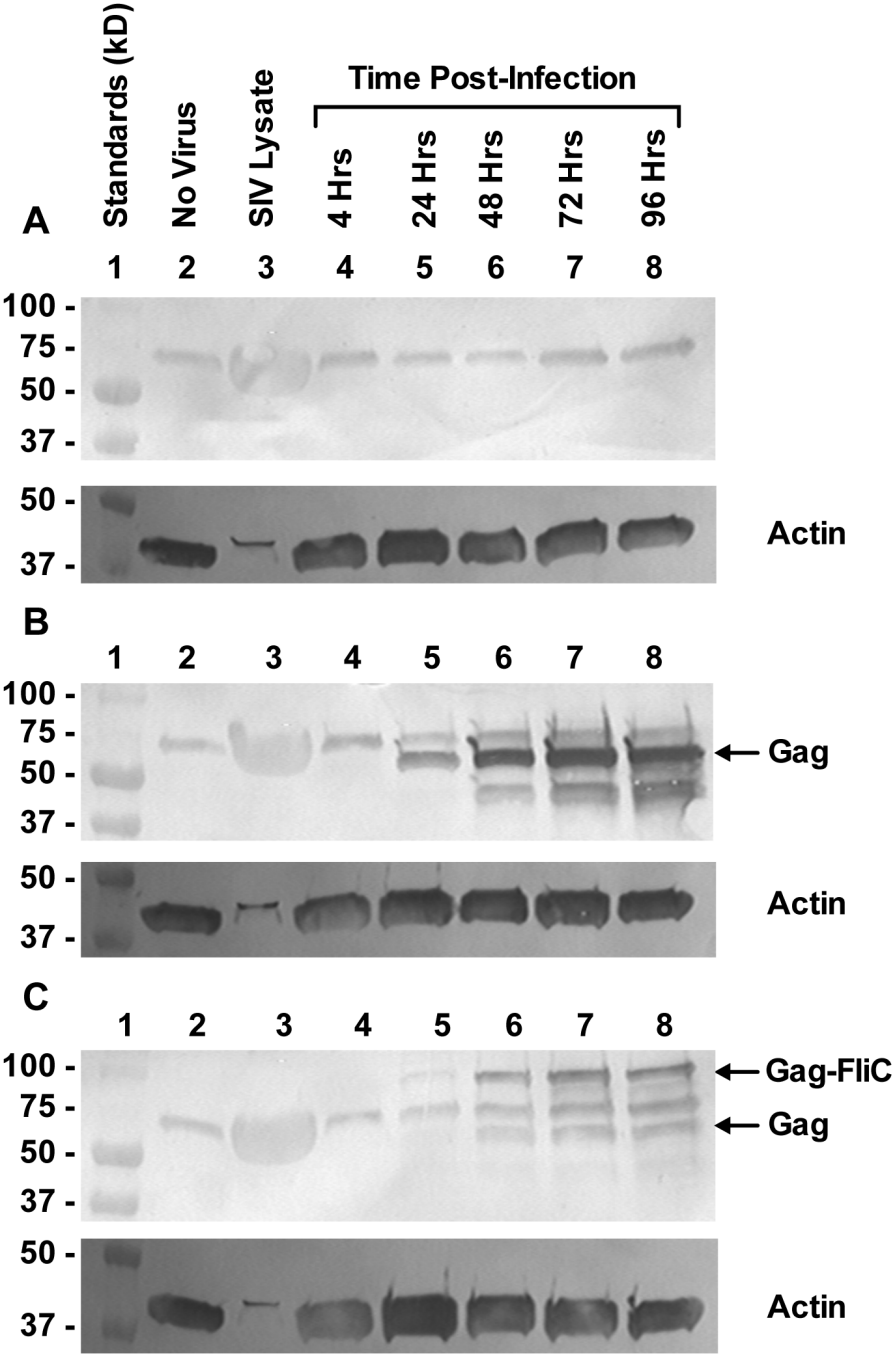
RhCMV*gagfliC*Δ196–378 expresses an SIV Gag-FliCΔ196–378 fusion protein. Western blot analysis of samples collected at the indicated times post-infection from TeloRF cells infected with either **(A)** RhCMV, **(B)** RhCMV*gag*, or **(C)** RhCMV*gagfliC*Δ196–378 were tested for reactivity with Gag p27 and actin (loading control) monoclonal antibodies. BioRad Precision Plus Protein Dual Color Standards indicate molecular weights (Lane 1). Lysates generated from TeloRF cells (No Virus; Lane 2) indicate background reactivity with the p27 antibody. Lysates of tissue culture supernatant collected from CEMx174 cells infected with SIVmac239 were used as positive controls for detection of SIV Gag (Lane 3).

To confirm stability of the Gag-FliCΔ196–378 fusion protein expressed from RhCMV*gagfliC*Δ196–378, infected TeloRF cell lysates were assessed for expression of SIV Gag by Western blot analysis. Cell lysates from TeloRF cells infected with RhCMV (Fig 7A), RhCMV*gag* (Fig 7B), or RhCMV*gagfliC*Δ196–378 (Fig 7C) were collected over 96 hours post-infection in denaturing sample buffer. Cell lysates (no virus) and all lysates generated from infected cells contained a background protein of approximately 70 kD that reacted with the SIV Gag (p27) monoclonal antibody (Lane 2 in Fig 7A–7C). TeloRF cells infected with RhCMV expressed no other SIV Gag (p27) antibody-reactive proteins. Lysates from RhCMV*gag*-infected TeloRF cells contained the expected 57 kD protein, equivalent to full-length SIV Gag polyprotein, and an additional unknown protein at approximately 40 kD, both of which increased in concentration over the course of infection. RhCMV*gagfliC*Δ196-378-infected TeloRF cell lysates contained the 57 kD SIV Gag protein, the unknown 40 kD protein, and another band at the expected 93 kD weight of the SIV Gag-FliCΔ196–378 fusion protein (854 amino acids). All three of these proteins (40 kD, 57 kD, and 93 kD) increased in concentration in the infected cells over time, peaking in concentration at approximately 72 hours post-infection. These results demonstrated that RhCMV*gagfliC*Δ196–378 in TeloRF cells expressed a fusion protein that cross-reacted with an SIV Gag (p27) monoclonal antibody at the appropriate size for a full-length SIV Gag-FliCΔ196–378 fusion protein.

### RhCMV expressing SIV Gag-FliC fusion proteins induce an inflammatory response *in vivo*

**Fig 8:**
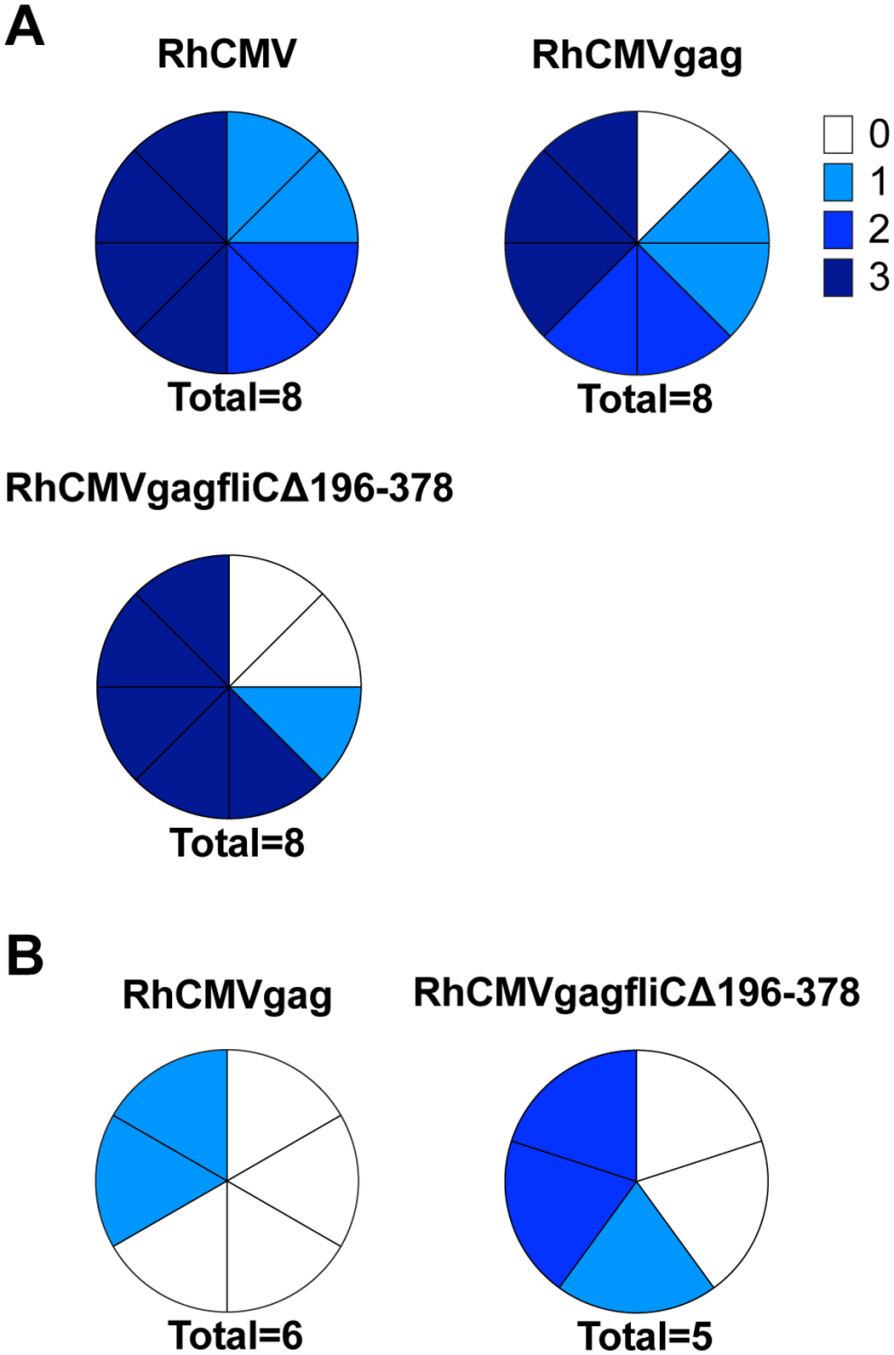
Inflammatory responses to RhCMV*gagfliC*Δ196–378 in RhCMV macaques. **(A)** Eight RhCMV sero-negative macaques were inoculated with RhCMV, RhCMV*gag*, or RhCMV*gagfliC*Δ196–378 and skin biopsies were collected seven days post-infection at the site of inoculation. **(B)** RhCMV sero-positive macaques were infected with either RhCMV*gag* (six macaques) or RhCMV*gagfliC*Δ196–378 (five macaques) and skin biopsies were collected at the site of inoculation seven days post-infection. Slides generated from the skin biopsies were stained by H&E and scored for inflammatory cell infiltrates. Charts indicate the observed inflammation with 0 (white) representing no observed inflammation, 1 (light blue) representing mild inflammation, 2 (medium blue) representing moderate inflammation, and 3 (darkest blue) representing severe inflammation.

Through stimulation of the TLR5 receptor, FliC induces the production of the proinflammatory cytokine TNF-α, resulting in the activation and recruitment of antigen presenting cells (APC). For this reason, we hypothesized that skin biopsies collected from macaques after subcutaneous (SC) inoculation with RhCMV expressing an SIV Gag-FliC fusion protein (RhCMV*gagfliC*Δ196–378) would demonstrate increased inflammatory cell infiltrates at the site of inoculation as compared to biopsies collected from macaques infected with the parental strain of RhCMV or RhCMV expressing only SIV Gag (RhCMV*gag*). To test this hypothesis, eight RhCMV seronegative macaques were inoculated SC with 1x10^4^ plaque forming units (pfu) of RhCMV68-1, RhCMV*gag*, or RhCMV*gagfliC*Δ196–378 and skin biopsies were collected seven days post-infection based on a previous study revealing this time point to show the optimum RhCMV expression in skin post SC infection [24]. Slides generated from the biopsies were stained with hematoxylin and eosin (H&E), and scored for inflammatory cell infiltrates (Fig 8A). Macaques from all three groups exhibited inflammatory responses at the site of inoculation that were characteristic of infection with RhCMV. Although the difference between groups was not statistically significant, macaques inoculated with RhCMV*gagfliC*Δ196–378 demonstrated increased inflammatory scores compared to macaques inoculated with RhCMV and RhCMV*gag*.

While CMV generates robust immune responses that control viral replication and prevent disease progression in immune competent hosts, there is evidence to suggest that these immune responses do not protect from re-infection with CMV. To determine whether RhCMV sero-positive macaques demonstrated the same trend towards increased inflammatory cell infiltrates at the inoculation site, six RhCMV seropositive macaques were inoculated with RhCMV*gag* and five RhCMV seropositive macaques were inoculated with RhCMV*gagfliC*Δ196–378. Slides generated from skin biopsies collected seven days post-inoculation were stained by H&E and scored for inflammatory cell infiltrates (Fig 8B). These biopsies exhibited a lower magnitude of inflammation overall compared to the biopsies collected from RhCMV sero-negative macaques (Fig 8A). However, the trend towards increased inflammation in RhCMV*gagfliC*Δ196–378 inoculated macaques observed in the sero-negative macaques was also observed in these RhCMV sero-positive macaques. Together, these data suggest that despite prior immune responses, RhCMV*gagfliC*Δ196–378 can induce pro-inflammatory responses at the site of inoculation that are unique to this RhCMV-vectored virus.

## Discussion

RhCMV vectors expressing lentiviral genes has demonstrated tremendous potential as a vaccine vector for HIV due to chronic antigenic stimulation and unique mechanisms of immune presentation as revealed by the RhCMV68-1 variant [11]. Here, we have described the generation of a recombinant RhCMV, RhCMV*gagfliC*Δ196–378, which encodes a novel fusion protein consisting of a truncated FliC, a TLR5 agonist, and SIVmac239 Gag. Results demonstrated that RhCMV*gagfliC*Δ196–378 is capable of stable expression of the SIV Gag-FliC fusion protein over multiple passages of virus as confirmed by western blot analysis and TLR5 bioactivity. Furthermore, expression of this fusion protein in RhCMV*gagfliC*Δ196-378-infected cells induces the pro-inflammatory cytokine TNF-α in a TLR5-dependent manner. Lastly, RhCMV*gagfliC*Δ196–378 induces a higher magnitude of inflammatory cell infiltrates in association with evidence of RhCMV expression and replication at the site of inoculation in rhesus macaques.

Flagellin has been investigated as an adjuvant in a growing body of studies (reviewed in [12]). While the majority of these studies utilize full-length FliC, FliCΔ196–378 encoding a partial truncation of the HV domain has been utilized as one approach to prevent the induction of an adaptive immune response to flagellin [14, 15]. The well-defined FliC TLR5-binding domains remained intact with this truncation and as a consequence, the deletion did not significantly affect TLR5 bioactivity (Fig 1B). Interestingly, a larger FliC truncation (FliCΔ141–398) demonstrated significantly reduced TLR5 bioactivity when compared to the FliCΔ196–378 truncation and wild-type FliC (Fig 1B). While the TLR5 binding domains within FliCΔ141–398 were predicted to remain intact based on a previous report [22], a different report showed that TLR5 binding utilized residues between 386–407 [25], which are affected by this 141–398 truncation. Due to a partial loss of TLR5 bioactivity, the FliCΔ141–398 truncation might prove useful in future studies designed to modulate levels of proinflammatory responses.

In this study, FliC was fused to the carboxyl-terminus of SIV Gag. This orientation was based upon previous studies and current literature (reviewed in [12]). Other orientations of FliC fusion proteins have been described, including N-terminal, C-terminal, and insertion of the antigen within the hypervariable domain of FliC [14, 15, 26]. While fusion proteins utilizing these various orientations have demonstrated efficacy, there is no current model to predict *a priori* the optimal orientation. Factors involved in optimal gene expression and adjuvant effects of FliC-antigen fusion proteins remain to be determined. It is possible that a stronger adjuvant effect might be obtained with an alternate SIV Gag-FliC fusion protein orientation. However, it is clear that the SIV Gag-FliC fusion protein described in this study induces TNF-α in a TLR5-specific manner, regardless of whether the protein is expressed from a plasmid or recombinant RhCMV vector. Moreover, expression of this fusion protein is stable over multiple passages of the RhCMV*gagfliC*Δ196–378 in cell culture.

The use of RhCMV as a lentiviral vaccine vector has been recently reviewed [27]. RhCMV vectors have demonstrated significant protection from disease progression in the macaque model of AIDS, with 50% of vaccinated animals able to control SIV replication after a mucosal challenge with pathogenic SIVmac239 [9, 10]. While RhCMV encodes numerous immune modulators, the inclusion of flagellin as a TLR5 agonist is unique. Human CMV [28] and RhCMV [29] encode an IL-10 homolog that reduces inflammatory responses at the site of inoculation [24]. Paradoxically, CMV can also induce proinflammatory cytokines and chemokines in infected monocytes and macrophages, including TNF-α [30–32]. A recent study demonstrated that infection of monocyte-derived macrophages with a low-passage clinical strain of HCMV resulted in the upregulation of several TLRs, including TLR5, and expression of NF-κB via the MyD88 signaling pathway [30]. Interestingly, that study also demonstrated that while infection of macrophages with HCMV induced the upregulation of TNF-α mRNA, treatment with lipopolysaccharide (LPS) was required for induction of TNF-α protein expression [30]. Another study utilizing a HCMV clinical isolate demonstrated that early in infection, HCMV down-regulates the TNF-α receptor [33], perhaps as a means to modulate TNF-α responses in order to tip the host-microbe balance in favor of infection. The long-term consequences of encoding a TLR5 agonist as an adjuvant within a high-passage fibroblast adapted RhCMV clone remain to be determined in ongoing experimental studies in rhesus macaques. However, our data clearly demonstrate that RhCMV*gagfliC*Δ196–378 replicates in cell culture with stable expression of the Gag-FliC fusion protein and also induces TNF-α in a TLR5-specific manner.

The use of FliC as an adjuvant for an HIV-1 vaccine has been previously described [34, 35]. One study utilized recombinant baculovirus to generate chimeric HIV virus-like particles containing membrane-bound HIV Envelope and HV domain-truncated FliC [34]. Enhanced systemic and mucosal immune responses in guinea pigs demonstrated the feasibility of FliC as an adjuvant for improving HIV-1 immune responses [34]. Another study utilized plasmid-encoded FliC fused to interleukin-18 as an adjuvant for SIV Gag, which was encoded on a separate plasmid [35]. BALB/C mice immunized with these plasmid vectors developed stronger immune responses to SIV Gag than animals vaccinated with plasmids encoding either IL-18 or FliC alone or delivered concomitantly [35]. However, these previous studies did not utilize a replicating vector.

This current study describes the construction and characterization of a novel replicating, RhCMV vector-based vaccine that expresses SIV antigen fused to a TLR5 ligand. The results described here demonstrate that the virus serves its function to induce TNF-α via TLR5. Experiments designed to determine whether this enhanced innate signaling will result in a more immunogenic and efficacious vaccine are ongoing and critical for evaluation of this type of adjuvant approach for an RhCMV-vectored SIV vaccine.

## Materials and Methods

### Experimental Animals and Infection

For the entirety of this study, 35 female rhesus macaques (Macaca mulatta; Mmu) were used from the retrovirus-free colony of the California National Primate Research Center (CNPRC) at the University of California (UC) at Davis. This study was approved by the UC Davis Institutional Animal Care and Use Committee (IACUC; approval number: 17880). UC Davis has an Animal Welfare Assurance on file with the NIH Office of Laboratory Animal Welfare (OLAW), and is fully accredited by the Association for the Assessment and Accreditation of Laboratory Animal Care, International (AAALAC). Animals were administered 10 mg/kg body weight ketamine-HCl (Parke-Davis, Morris Plains, NJ, USA) intramuscularly when necessary for immobilization. Additionally, analgesics were administered at the discretion of the CNPRC veterinary staff in an effort to minimize pain and discomfort. Animals were maintained at the CNRPC in cages with 4 square feet of floor space, or 6 square feet if over 10 kg, with fixed perch bars in a temperature-controlled BSL-2+ vivarium with continuous monitoring of temperature and humidity. Compatible animals were paired continuously or intermittently (separated at night) whenever possible. All animals had visual and auditory access to other macaques 24 hours per day. These animals were fed a balanced commercial macaque chow (Purina Mills, Gray Summit, MO) twice daily and fresh produce twice weekly, with free access to water 24 hours per day. Supplemental food was provided when clinically indicated. Environmental enrichment was provided daily, including manipulanda (forage boards, mirrors, puzzle feeders) and novel foodstuffs. All animals are involved in ongoing studies designed to determine vaccine efficacy and remain housed as described.

Each macaque was inoculated subcutaneously (SC) in the back with 1x10^4^ plaque forming units of either: RhCMV (68–1), RhCMVgag, or RhCMV*gagfliC*Δ196–378. Skin biopsies were taken one week post-inoculation and the animals were returned to their housing.

### Construction of FliC Expression Vectors

#### Full-length FliC expression vector

Plasmid pET29a*flagellin* encoding *Salmonella enterica* serovar Enteritidis FliC (*flagellin*) (kindly provided by S. Mizel; Wake Forest University) was used to construct a flagellin eukaryotic expression vector [36]. *fliC* was cloned into the eukaryotic expression plasmid pSV40EC1-72, generating plasmid pSV40*fliC* using standard molecular biology techniques. Plasmid clones were submitted to UC Davis Sequencing for verification. Plasmid pORI, containing an R6K origin of replication, was acquired from Louis Picker (Oregon Health Sciences University). *fliC* from pSV40*fliC* was cloned into pORI using standard molecular biology techniques, generating plasmid pORI*fli*C. Plasmid clones were propagated in *E*. *coli* DH5α-λpir+ and submitted to UC Davis Sequencing for verification.

#### FliC hypervariable region mutant expression vectors

Overlap-extension PCR [37] was used to truncate the hypervariable (HV) domain of FliC. To generate FliCΔ196–378, nucleotides 586 to 1134 encoding amino acids 196 to 378 were deleted (GenBank: AY353386) [14]. A second FliC HV region truncation mutant was generated by deleting nucleotides 424 to 1194 encoding amino acids 141 to 398 generating FliCΔ141–398. Primers for the first round PCR amplification of *fliC*Δ196–378 construct were WLC81 and JDD4 for nucleotides 1 through 423 and JDD3 and WLC82 for nucleotides 1135 through 1518 (Table 1). Primers for the first round PCR amplification of the *fliC*Δ141–398 construct were WLC81 and JDD4 for nucleotides 1 through 423 and JDD3 and WLC82 for nucleotides 1135 through 1518 (Table 1). Round two PCR was performed using round one PCR products as template and primers WLC81 and WLC82 (Table 1). Round two PCR products were cloned into the AgeI and NotI REase sites of the eukaryotic expression plasmid pSV40EC1-72, generating plasmids pSV40*fliC*Δ196–378 and pSV40*fliC*Δ141–398. *fliC*Δ196–378 and *fliC*Δ141–398 were subsequently cloned into the BamHI and SalI REase sites of plasmid pORI, generating pORI*fliC*Δ196–378 and pORI*fliC*Δ141–398. Plasmid clones were propagated in *E*. *coli* DH5α-λpir+ and submitted to UC Davis Sequencing for verification.

**Table 1:** PCR Primers.

Name	Sequence*
WLC81	AAACCGGTCGCCACCATGGCACAAGTCATTAATAC
WLC82	TTGCGGCCGCTTAACGCAGTAAAGAGAGG
JDD1	AAAGTCCTGTCTCAGGACGGCGTAAGTACATTAATC
JDD2	GATTAATGTACTTACGCCGTCCTGAGACAGGACTTT
JDD3	TTCAAGAATGTTACGGGTGCCACGGGTGATAAGATC
JDD4	GATCTTATCACCCGTGGCACCCGTAACATTCTTGAA
JDD7	AAGCTAGCCGCCACCATGGG
JDD8	AAACCGGTCTGGTCTCCTCCAAAGAGAGAATTG
JDD9	AAACCGGTGCACAAGTCATTAATACAAACAG
JDD10	AAGCGGCCGCGTCGACTTAACGCAGTAAAGAGAGG

*All sequences are written 5’ to 3’

#### SIV Gag-FliC fusion protein expression vectors

To generate an SIV Gag-FliC fusion protein expression vector, *fliC* was PCR amplified from pET29a*flagellin* using primers JDD9 and JDD10 (Table 1) as described above. These primers were designed to delete the Kozak sequence and the start codon as well as to introduce a novel AgeI REase site at the 5’ end and a novel SalI REase site at the 3’ end of *fliC*. The final PCR product was cloned into pCR2.1-TOPO (Invitrogen), generating the plasmid pCR2.1*fliC*(ΔATG). *fliC*Δ196–378 was also prepared for construction of an SIV Gag fusion protein using overlap-extension PCR as described above to truncate the hypervariable region of FliC, delete the Kozak sequence and the start codon, and to introduce AgeI and SalI REase sites. The first round primer pairs were JDD9/JDD4 and JDD3/JDD10 (Table 1). The second round primers were JDD9 and JDD10 (Table 1). The final PCR product was cloned into pCR2.1-TOPO (Invitrogen), generating p2.1*fliC*Δ196-378(ΔATG). The plasmid pORI*gag*, encoding a codon-optimized SIVmac239 Gag, was acquired from Louis Picker (OHSU). SIV*gag* was PCR amplified using primers JDD7 and JDD8 (Table 1). The primers were designed to introduce a novel NheI REase site upstream of SIV*gag*, a novel AgeI REase site at the 3’ end, and to remove the downstream flag tag and stop codon. The PCR product was cloned into pCR2.1-TOPO (Invitrogen) to generate pCR2.1*gag*. Due to SIV*gag* subcloning orientation problems, possibly resulting from bacterial selection due to read-through from the *lac*Z gene of pCR2.1-TOPO, *fliC*(ΔATG) and *fliC*Δ196-378(ΔATG) were cloned into the EcoRI and SalI REase sites of pUC19, generating the plasmids pUC19*fliC*(ΔATG) and pUC19*fliC*Δ196-378(ΔATG). Next, SIV*gag* from pCR2.1*gag* was cloned into the EcoRI and AgeI REase sites of the pUC19*fliC* vectors, generating the plasmids *pUC19gagfliC* and pUC19*gagfliC*Δ196–378. *gagfliC* and *gagfliC*Δ196–378 were then cloned into the NheI and SalI REase sites of the pORI expression vector, generating plasmids pORI*gagfliC* and pORI*gagfliC*Δ196–378. These plasmids encoded a SIV*gag* gene separated from *fliC* by a single AgeI REase site, a single Kozak sequence and start codon at the start of SIV*gag*, and a single stop codon at the 3’ end of *fliC*. The plasmids were propagated in *E*. *coli* DH5α-λpir+ and submitted to UC Davis Sequencing for verification.

### FliC TLR5 Functional Assay

The TLR5-specific functional activity of FliC, FliCΔ196–378, and FliCΔ141–398 expression plasmids were tested by measuring TNF-α production *in vitro* using an ELISA as previously described [14]. Telomerized rhesus fibroblast cells (TeloRFs) [38] were seeded at 5 x 10^5^ cells per well 2 ml of DMEM supplemented with 10% Fetal Bovine Serum (FBS) and 1X penicillin plus streptomycin in 6-well tissue culture plates and incubated overnight at 37°C. The TeloRFs were then transfected with pORI*fliC*, pORI*fliC*Δ196–378, or pORI*fliC*Δ141–398 using FuGENE6 transfection reagent (Promega) according to the user’s manual and returned to the 37°C incubator. The transiently transfected TeloRFs were collected by removing growth media, washing the cells once with 1X phosphate buffered saline (PBS), adding 500 μl of growth media, lifting the cells from the tissue culture well with a rubber cell scraper and were then transferred to 1.5 ml centrifuge tubes. Lysates were generated from freezing the cell suspensions at -80°C, thawing in a water bath at 37°C, and repeating for a total of three freeze/thaw cycles. Lysates were then repeatedly passed through a 22-gauge needle attached to a 1 ml syringe and stored at -20°C. Recombinant FliC and the mouse macrophage cell lines RAW264.7 (TLR5 negative) and RAW424 (TLR5 positive) [39] were acquired from Steven Mizel (Wake Forest University) and used to test the lysates from FliC-transfected TeloRFs for TLR5 activity as previously described [14]. Briefly, 4 x 10^5^ RAW264.7 cells and 5 x 10^5^ RAW424 cells were seeded in 1 ml of growth media per well in 24-well plates and incubated at 37°C overnight in a humidified CO2 incubator. The cells were then treated with 20 μl of lysate from TeloRFs or with recombinant FliC for four hours at 37°C. Cell culture supernatant was collected and stored at -20°C until analysis for TNF-α content by ELISA (UCytech) according to the manufacturer’s instructions. Preliminary experiments demonstrated maximal TNF-α production on RAW424 cells with treatment of FliC-transfected TeloRF lysates collected between 24 and 48 hours post-transfection; the 48-hour time point was subsequently used throughout the remainder of the study.

### RhCMV Expressing SIV*gagfliC*Δ196–378

To generate a recombinant RhCMV that expresses the SIV*gagfliC*Δ196–378 fusion protein, Red/ET recombination (Gene Bridges) [40] was used as previously described [8] to insert SIV*gagfliC*Δ196–378 from the plasmid pORI*gagfliC*Δ196–378 into the non-coding region between rh213 and rh214 of a bacterial artificial chromosome (BAC) containing a complete molecular clone of the RhCMV 68–1 genome, RhCMV-*loxP*(r) [23]. Briefly, a culture of DH10B *E*. *coli* carrying RhCMV-*loxP*(r) encoding chloramphenicol (Cam) resistance and plasmid pSC101-BAD-gbaA containing a temperature sensitive origin of replication and encoding tetracycline (Tet) resistance [40] was propagated overnight at 30°C in LB broth containing Cam (25 μg/ml) and Tet (3 μg/ml). The overnight stationary culture containing selective antibiotics was propagated at 30°C until an optical density at 600 nm of 0.2. L-arabinose was added to 0.2% to induce expression of the recombinases, and the cells were propagated at 37°C until an optical density at 600 nm of 0.3–0.4. A PCR product containing the EF1-α promoter, SIV*gagfliC*Δ196–378, and a kanamycin (Kan) resistance cassette was generated from the PCR amplification of pORI*gagfliC*Δ196–378 with Advantage 2 DNA polymerase (Clonetech) using primers WLC87 (5’ GGGAAATCACGTCATCAGGCTGGGTAGTCAACATGGGCATACGAAACTTGCCCGAATAGATGCTCTCACTTAACGGCTGACATG 3’) and WLC88 (CCAGAATGTGCTCTACTTTTTGGCCAGCGGGTTGGATGATTTCGCGCGTCATGGACTGCTTCACTGTAGCTTAGTACGTTAAAC). The purified PCR product was transformed into the recombination-ready electrocompetent DH10B *E*. *coli* using electroporation in a Gene Pulser (BioRad) and transformed cells were recovered in SOC media for 2–3 hours at 37°C, plated on LB agar containing Cam (25 μg/ml) and Kan (30 μg/ml) and incubated overnight at 37°C. Single colonies were selected and grown overnight in LB broth containing Cam and Kan at 37°C. BAC DNA was isolated from the cultures using the Boil Prep Procedure and precipitation in isopropanol. The purified BAC DNA was then screened by PCR for the presence of the EF1-α promoter-SIV*gagfliC*Δ196-378-Kan resistance cassette insert in between the Rh213 and 214 region of the RhCMV genome using primers JDD19 and JDD8 specific for Rh213 and SIV*gag*, respectively, and primers JDD9 and JDD20, specific for *fliC* and Rh214. Clones were selected and propagated in LB broth containing Cam (25 μg/ml) and Kan (30 μg/ml). Cells were transformed with pCP20, containing a temperature-sensitive origin of replication and expressing *flp* recombinase and a *bla* antibiotic resistance gene, by electroporation. After recovery, cells were plated on LB agar plates containing Cam (25 μg/ml) and Amp (50 μg/ml) and incubated overnight at 30°C. Selected colonies were streaked onto LB agar plates containing Cam (25 μg/ml) and incubated overnight at 43°C to induce *flp* recombinase. Colonies were selected from the 43°C plates and screened for Amp and Kan sensitivity. Selected clones were propagated in LB broth containing Cam (25 μg/ml) and BAC DNA was isolated using the boil prep method. Purified BAC DNA was analyzed by PCR for presence of the EF1-α promoter-SIV*gagfliC*Δ196–378 insert in between Rh213 and Rh214 of the RhCMV genome in addition to the absence of the Kan resistance gene using primer pairs JDD19/JDD8 and JDD9/JDD20 as described above. Positive clones were selected, and BAC DNA was purified using a NucleoBond BAC Maxi kit (BD Biosciences) according to user’s manual.

The accuracy of the insert was further confirmed through a primer walking and Sanger sequencing strategy. The entire region of approximately six kilobases was PCR amplified with primer pair prRh90-F/prRh90-R and the amplicon was Sanger sequenced with the primers listed in Table 2. The sequence and stability of the insert was also investigated with high throughput sequencing of the purified BAC on the ION Torrent platform, either by direct sequencing or PCR amplifying the entire construct. Library preparation, sequencing and analysis were performed as described previously [41]. High throughput sequencing of the construct directly or after PCR amplification resulted in near 100% coverage of the RhCMV genome with low error rates (S1 Fig). The insert sequence was highly stable in the sequenced pool of BAC constructs, with no evidence of low frequency insertions or deletions within the region.

**Table 2:** Sequencing Primers.

Name	Sequence
prRh90-F	GAACCATGTAGTTTTCACGAG
prRh90-R	TGACATGAAGGGCAATAAAGC
prRhInsF-Seq1	TGGGCATCCTTCTAAGATGG
prRhInsF-Seq2	CCATATGGAATAACACCGAAC
prRhInsF-Seq3	TCCATGTACAGATAGCGGTC
prRhInsF-Seq4	CGTATATAAGTGCAGTAGTCG
prRhInsF-Seq5	CGAGCTTTTGGAGTACGTCG
prRhInsR-Seq1	GAATAGGAACTTCTGAATTCGAC
prRhInsR-Seq2	CGTGGCACCCGTAACATTC
prRhInsR-Seq3	TTCTTTAGCAGATCCACAGC
prRhInsR-Seq4	GTCTACATAGCTCTGAAATGG

TeloRFs were transfected with BAC DNA using FuGENE (Roche) according to the user’s manual and were maintained in culture until apparent cytopathic effect. Cell culture supernatant was collected and used to infect fresh TeloRFs. Virus that was serially passed through three passages was expanded in TeloRFs, purified and stored in liquid nitrogen vapor as previously described [23]. Lysates generated from TeloRF cell pellets during serial passage were tested for FliC TLR5 functional activity as described above.

### Western Blot Analysis

For SIV Gag Western blot analysis, TeloRF cell lysates were separated by SDS-PAGE and transferred to polyvinylidene fluoride (PVDF) transfer membranes (Amersham) using standard laboratory procedures. The membranes were blocked for nonspecific protein binding overnight at room temperature in 5% skim milk and 0.1% Tween20 in PBS and then probed using SIV Gag p27 monoclonal antibody [42]. An anti-mouse IgG peroxidase conjugated antibody (Sigma clone: A5906) and 3,3’-diaminobenzidine (DAB; Vector Laboratories) were used for immunodetection. Western blot analysis for β-Actin (C4) (Santa Cruz Biotechnology; sc-47778 HRP) was also performed on the TeloRF cell lysates according to the manufacturer’s instructions.

### Histopathology

Prior to the collection of biopsies, animals were sedated with Ketamine and 0.2 mL of Lidocaine was administered to the biopsy site to minimize discomfort according to an IACUC-approved protocol (FF-1). The biopsy site was shaved and the area was cleansed using betadine scrub solution, alcohol, and betadine prep solution by a sterile saline rinse. Biopsies were obtained using a sterile, six mm punch type biopsy tool with the help of a sterile scalpel blade if needed. Post-exposure analgesics were administered according to an IACUC-approved protocol (LL-5). Biopsies were fixed in neutral buffered formalin, and processed by routine histology technique. Sections were stained with hematoxylin and eosin, coded with random numbers, and then blindly examined by a board-certified veterinary pathologist (SWB).

Although the biopsies all represented areas of inoculation, the depth of the inoculation was variable, ranging from dermis to subcutis and panniculus carnosis. Some of the samples did not contain all tissue layers. The nature of the inflammation was similar among all positive samples, but was multifocal and varied in location and depth within the sample. Therefore, it was not possible to comparatively score the samples by cell count or other objective criteria. They were therefore scored on an overall scale of 0 (no inflammation present) to 3 (severe inflammation). Inflammation was independently scored for the dermis, subdermis, and subcutis/panniculus carnosis, and the overall highest score was used for comparison among samples (S2–S5 Figs). Mild inflammatory changes (1+) consisted of focal perivascular infiltrates of lymphocytes, plasma cells, few polymorphonuclear cells, and macrophages, some of which were enlarged (cytomegaly) and contained intranuclear viral inclusion bodies. Moderate inflammatory changes (2+) consisted of multifocal areas of similar cellular infiltrates with evidence of vascular hyperemia, and edema. Severe lesions (3+) had intense multifocal and often coalescing areas of leukocyte infiltration and hemorrhage with numerous cytomegalic macrophages with intranuclear viral inclusion bodies. Focal acute hemorrhage, associated with surgical excision, was present in most biopsies.

## Supporting Information

S1 Fig Cumulative error probability from RhCMV BAC resequencing data. The entire RhCMV BAC was either directly sequenced (red) or sequenced after PCR amplification (blue). Polymorphisms within the data were assumed to be sequencing errors, allowing for estimation of error rates. The cumulative error probability from these data was then plotted.
(PDF)

S2 Fig Representative Image of Inflammatory Score 0. This is a representative image of a skin biopsy corresponding to an inflammatory score of 0 (no inflammation present).
(TIF)

S3 Fig Representative Image of Inflammatory Score 1. This is a representative image of a skin biopsy corresponding to an inflammatory score of 1 (mild inflammation present).
(TIF)

S4 Fig Representative Image of Inflammatory Score 2. This is a representative image of a skin biopsy corresponding to an inflammatory score of 2 (moderate inflammation present).
(TIF)

S5 Fig Representative Image of Inflammatory Score 3. This is a representative image of a skin biopsy corresponding to an inflammatory score of 3 (severe inflammation present).
(TIF)
