# Supplementary figures and images for: Utilizing a TLR5-Adjuvanted Cytomegalovirus as a Lentiviral Vaccine in the Nonhuman Primate Model for AIDS
Source: PLoS One. 2016 May 16;11(5):e0155629. doi: 10.1371/journal.pone.0155629 (PMC4868283; doi:10.1371/journal.pone.0155629)

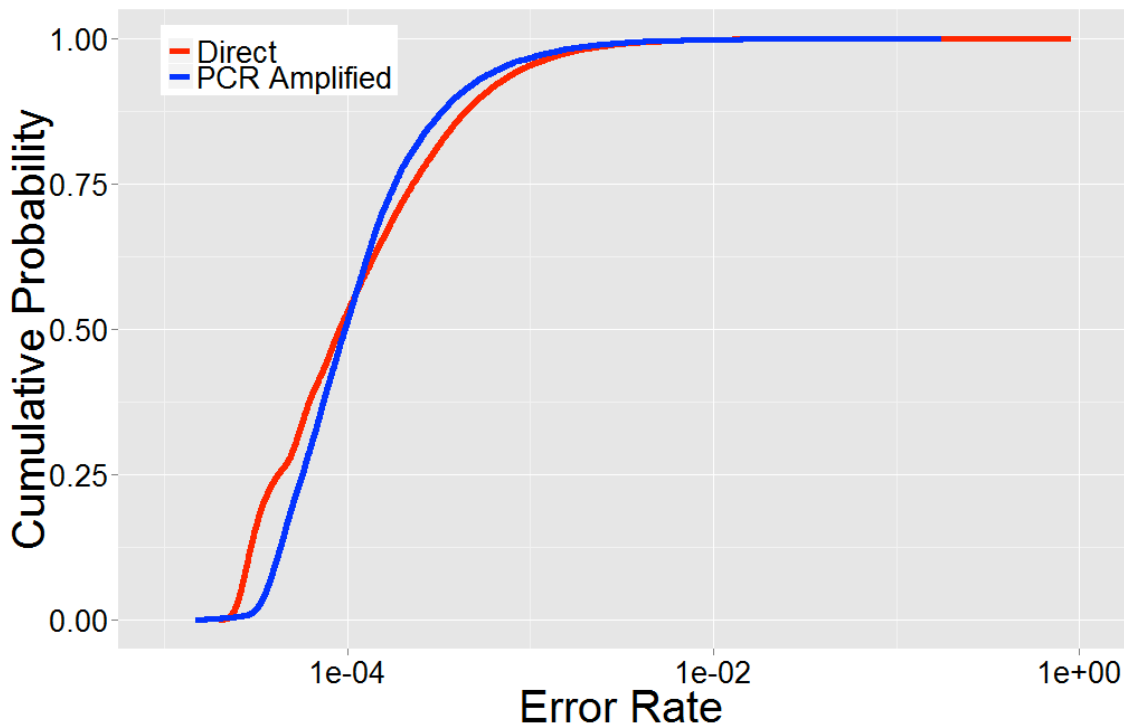

Supplement: S1 Fig — The entire RhCMV BAC was either directly sequenced (red) or sequenced after PCR amplification (blue). Polymorphisms within the data were assumed to be sequencing errors, allowing for estimation of error rates. The cumulative error probability from these data was then plotted. (PDF) [file pone.0155629.s001.pdf]

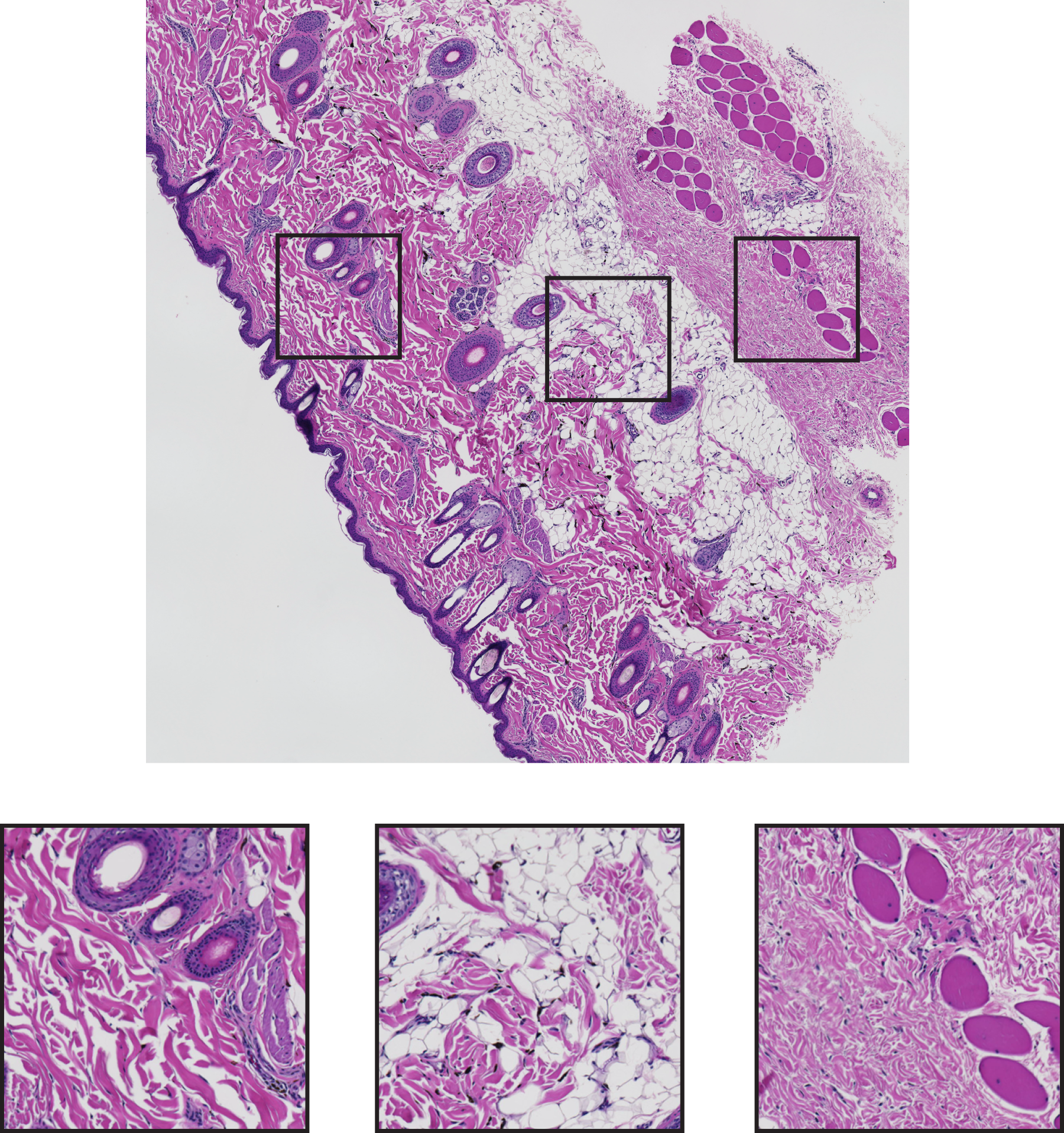

Supplement: S2 Fig — This is a representative image of a skin biopsy corresponding to an inflammatory score of 0 (no inflammation present). (TIF) [file pone.0155629.s002.tif]

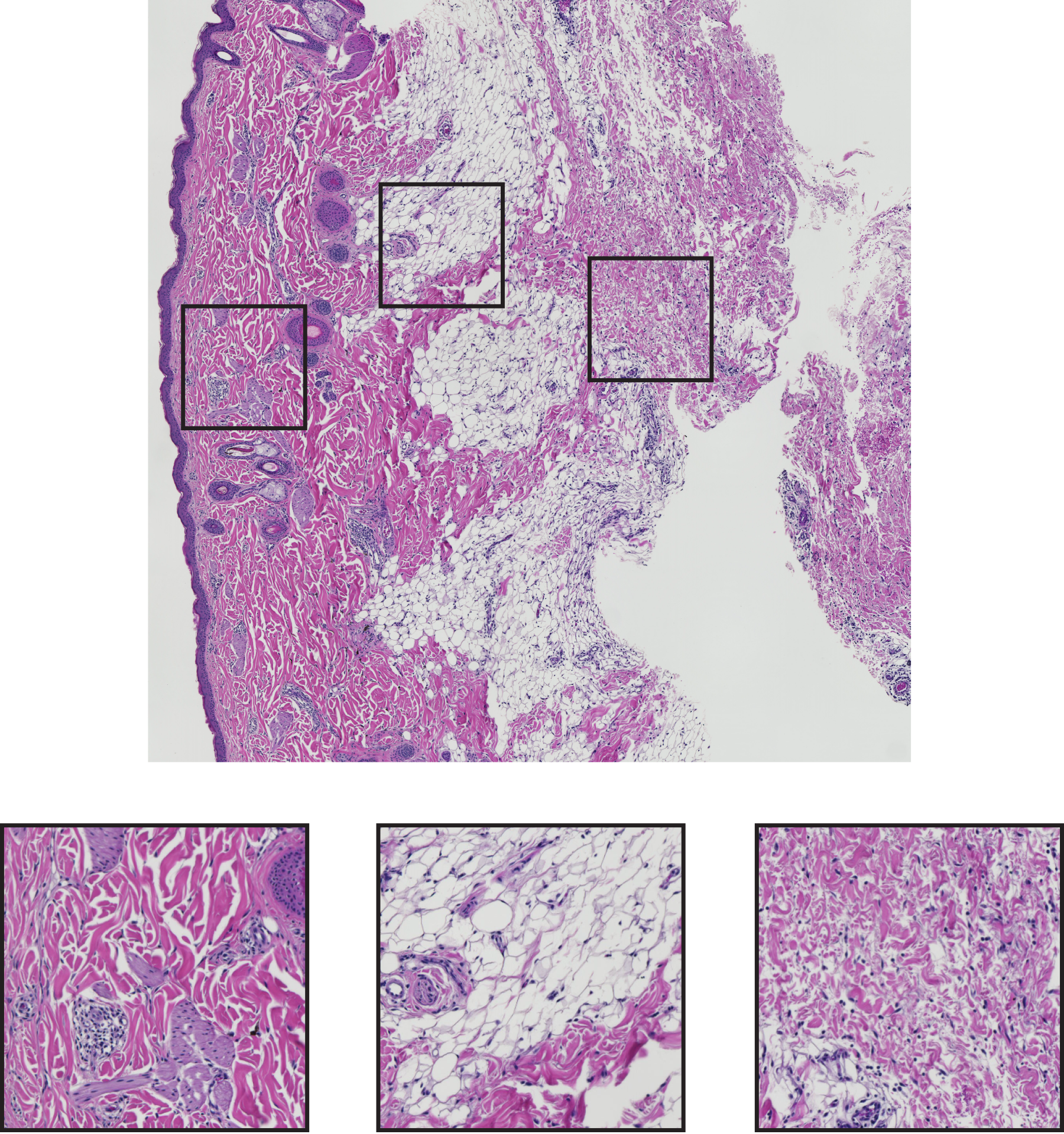

Supplement: S3 Fig — This is a representative image of a skin biopsy corresponding to an inflammatory score of 1 (mild inflammation present). (TIF) [file pone.0155629.s003.tif]

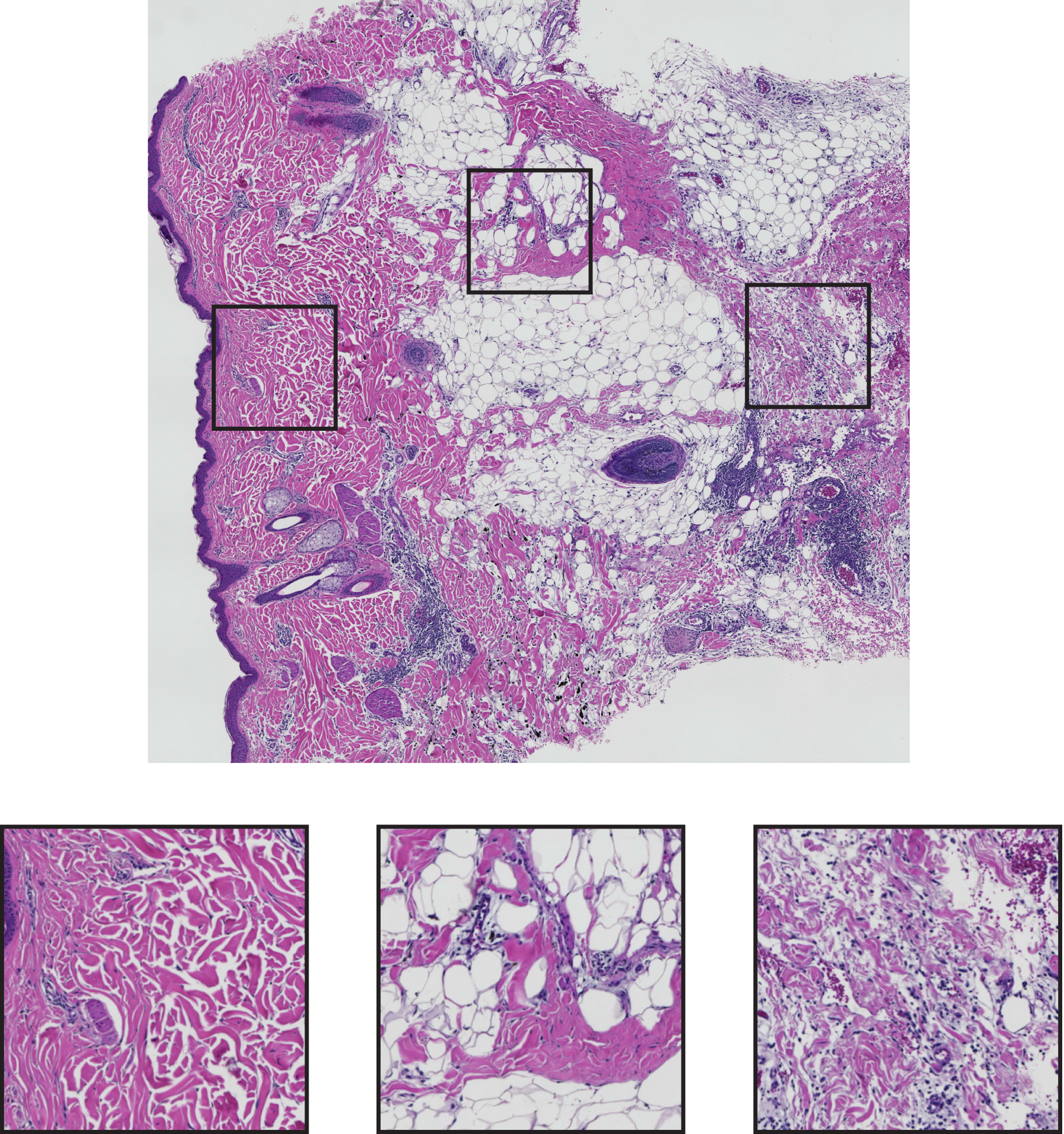

Supplement: S4 Fig — This is a representative image of a skin biopsy corresponding to an inflammatory score of 2 (moderate inflammation present). (TIF) [file pone.0155629.s004.tif]

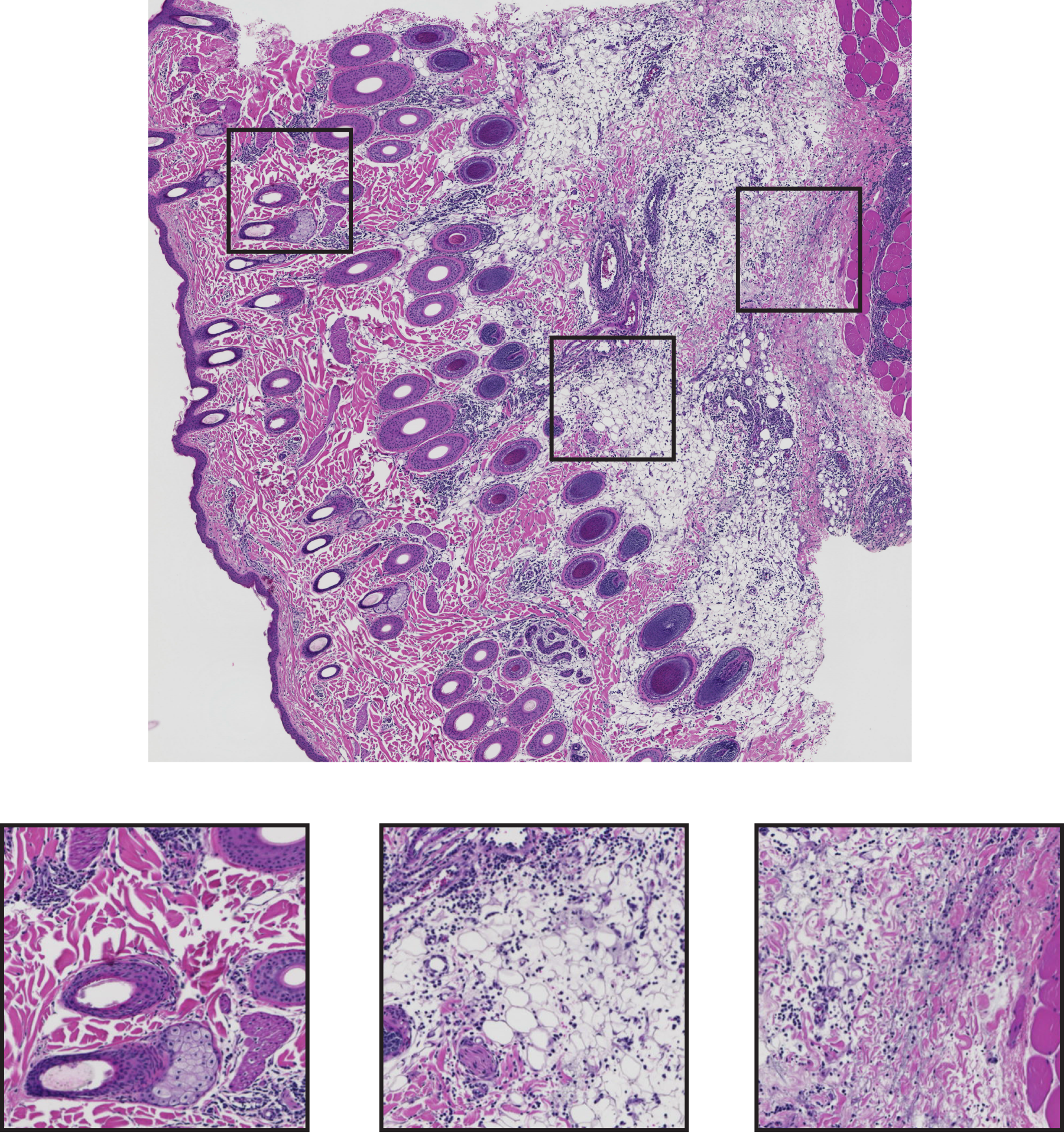

Supplement: S5 Fig — This is a representative image of a skin biopsy corresponding to an inflammatory score of 3 (severe inflammation present). (TIF) [file pone.0155629.s005.tif]
